# Effects of Cyclic Aeration Subsurface Drip Irrigation on Greenhouse Tomato Quality and Water and Fertilizer Use Efficiency

**DOI:** 10.3390/plants13243559

**Published:** 2024-12-20

**Authors:** Zhongqiu Liu, Kaidi Long, Jian Zeng, Yan Zhang, Qinghua Shi, Bing Hui, Peng Zhang, George Papadakis, Qian Zhang

**Affiliations:** 1 College of Water Conservancy and Civil Engineering, Shandong Agricultural University, Tai’an 271018, China; zqliu08@sdau.edu.cn (Z.L.); sdaulkd@163.com (K.L.); zj19970709@163.com (J.Z.); 13021532521@163.com (B.H.); tazhangpeng@163.com (P.Z.); 2 Digital Twin Agricultural Technology Research Center, Institute of Smart Agriculture, Shandong Agricultural University, Tai’an 271018, China; gpap@aua.gr; 3 College of Horticulture Science and Engineering, Shandong Agricultural University, Tai’an 271018, China; zhangyan2022@sdau.edu.cn (Y.Z.); qhshi@sdau.edu.cn (Q.S.); 4 Department of Natural Resources and Agricultural Engineering, Agricultural University of Athens, Iera Odos 75, 11855 Athens, Greece

**Keywords:** greenhouse tomatoes, cyclic aeration underground drip irrigation, quality, water use efficiency, fertilizer utilization efficiency

## Abstract

Tomato (Jinglu 6335) was selected for assessing the impact of varying fertilizer (F:N-P_2_O_5_-K_2_O) and aeration rates on crop quality, as well as water and fertilizer utilization efficiency during the cyclic aeration subsurface drip irrigation process. Four aeration treatments (O1, O2, O3, and S, representing aeration ratios of 16.25%, 14.58%, 11.79%, and non-aerated treatment, respectively) and three fertilizer applications (F1: 240–120–150 kg/hm^2^, F2: 180–90–112.5 kg/hm^2^, F3: 120–60–75 kg/hm^2^) were compared in a total of 12 treatments in this study. This study revealed that cyclic aerated drip irrigation improved the fruit quality. The aerated treatment resulted in increased accumulation of nitrogen, phosphorus, and potassium, with the level of aeration positively correlating with the increase in nutrient accumulation, reaching the highest values in the high aeration irrigation treatment. The highest nitrogen, phosphorus, potassium, and water use efficiency occurred under the medium fertilizer with high aeration treatment. The maximum partial productivity of the fertilizer occurred under the low fertilizer with high aeration treatment, while the minimum occurred in the high fertilizer with non-aerated treatment. Taking all factors into consideration, the high-aeration and medium-fertilizer treatment was the most effective combination for greenhouse tomatoes under the conditions in this experiment.

## 1. Introduction

Crops absorb water and nutrients in the processes of growth and development [1,2], which requires roots to supply oxygen. The oxygen content in the soil directly impacts crop root respiration and nutrient absorption, subsequently influencing crop growth and development [3,4]. In modern agricultural practices, subsurface drip irrigation is widely recognized as a water-saving technique [5]. However, after irrigation, the soil becomes saturated with water, causing the gas to be forcibly discharged, which can inhibit crop root respiration, impede crop growth and development, and ultimately reduce both the yield and quality of crops [6,7].

Pendergast et al. found that the oxygen diffusion rate near the drip head in subsurface drip irrigation is at its lowest, and prolonged irrigation can result in soil hypoxia near the drip head [8]. Machado et al. discovered that crop roots tend to be concentrated around the emitter due to water absorption in the process of subsurface drip irrigation. Consequently, crop roots are frequently exposed to an environment with low oxygen levels [9]. Payero et al. found that soil hypoxia leads to a reduction in crop yield [10]. Takeshi discovered that inadequate soil oxygen in the root zone restricts the energy supply to root cells, diminishes root activity, impedes the absorption of water and nutrients, and ultimately results in insufficient dry matter accumulation in crops [11].

Aerated drip irrigation uses venturi aerating equipment to inject air into a subsurface drip irrigation stream, facilitating the transportation of both irrigation water and air to the soil around the crop rhizosphere [12]. Previous research has demonstrated that aeration drip irrigation can enhance crop yield and water use efficiency, ultimately fostering crop growth and increasing overall yield [13,14,15,16]. Chen et al. conducted a study on the impact of aeration volume and irrigation depth on cotton, which indicated that at the same irrigation depth, aerated treatment led to a 14% increase in cotton yield. Furthermore, the quality of dry matter and water use efficiency were significantly improved compared to the control treatment [17]; Bhattarai et al. conducted a survey on the impact of aerated irrigation and the burial depth of drip irrigation on a crop, and the results showed that oxygenation had the greatest effect on the yield of a shallow-root crop kidney bean (+43%), while it had a minimal impact on the middle-root crop chickpea (+11%) and deep-root crop pumpkin (+15%). Aerated irrigation invariably increased water use efficiency, biomass yield and instantaneous leaf transpiration rate [7]. 

Niu et al. conducted an aerated irrigation test on maize under different irrigation regimes. The results indicated that aerated irrigation improved plant height, leaf area, and root activity compared to non-aerated irrigation under the same regime. Specifically, during the jointing period with an irrigation regime of 600 mL and aeration every 4 days, root activity increased by 66.7% [18]. Chen et al. reported that aerated drip irrigation could lead to a 17.3% increase in pineapple yield and a 4.3% improvement in water use efficiency [19]. There is an interactive effect between fertilization and aeration irrigation on the fruit quality and yield of greenhouse tomatoes. Yang et al. found that nitrogen fertilizer application affected the quality of pomegranate fruit, aril and juice, and increasing the concentration of nitrogen fertilizer application helped to increase the ratio of edible aril weight to total fruit weight [20]. Li et al. explored the effects of the coupling of ventilation and fertilization on the photosynthesis, chlorophyll content, yield, and quality of tomatoes. They concluded that fertilization improved the photosynthetic performance of the plants, and the combined application of these factors enhanced both the yield and quality of tomato fruits [21]. Sun et al. found that irrigation, fertilization, and aeration treatments affected the organic acid, soluble sugar, and lycopene contents of tomato fruits [22]. 

Previous research predominantly focused on factors such as aeration volume, irrigation regimes and the burying depth of subsurface drip irrigation, with a primary emphasis on water use efficiency, crop yield, and quality [23,24]. Information on fertilizers absorption and utilization with aerated irrigation is limited. The objective of this study was to investigate the efficiency of water and fertilizer utilization, as well as fruit quality, in order to ultimately explore optimal aeration and fertilizer coupling for greenhouse tomatoes. The study hypothesized that aerated irrigation could promote nutrient accumulation and fruit quality, and help improve water and fertilizer use efficiency.

## 2. Materials and Methods

### 2.1. Site and Crop Description

This experiment was carried out in a Chinese-type solar greenhouse at Shandong Agricultural University (36°18′ N, 117°17′ E). The solar greenhouse measured 5.5 m in height, 13 m in width, and 52 m in length from east to west. The effective planting area within the greenhouse was 450 square meters. The experimental area was located in a temperate continental semi-humid monsoon region characterized by an annual average greenhouse temperature of 20 °C and an average annual sunshine duration of 2627.1 h. The soil type was classified as brown loam, with available nitrogen at 136.5 mg/kg, available phosphorus at 51.72 mg/kg, and available potassium at 168.07 mg/kg. The pH and EC values were 6.5 and 0.62 mS/cm, respectively. The conductivity of the irrigation water was 671 μs/cm, the pH value was 7.2, the TDS value was 336 mg/L, the dissolved oxygen content was 6.06 mg/L, the CO_3_^2−^ concentration was 0 meq/L, and the HCO_3_^−^ concentration was 3.14 meq/L.

The experimental tomato variety was Jinglu 6335, which belongs to the category of medium- and late-maturing varieties and is one of the main cultivars grown in Shandong Province. The inner ridge of the solar greenhouse measured 10 m in length and 0.8 m in width, separated by a plastic film with a buried depth of 100 cm from the exterior to prevent lateral infiltration, with one ridge assigned to each treatment. The diameter of the drip irrigation pipe was 16 mm, the designed flow of the emitter was 2 L/h, the spacing of emitter was 33 cm, and the maximum working pressure was 0.2 MPa. The plants were arranged based on the spacing of the drippers, with a horizontal distance of approximately 10 cm between each treatment and a buried depth of 25 cm. the water supply pipeline of each treatment was independently controlled and equipped with a precision water meter. All field management practices for the treatments, encompassing fertilizer application, irrigation, and pesticide spraying, strictly adhered to the methods employed by local farmers and were maintained as consistently as possible.

### 2.2. Experimental Design

The experiment had two irrigation treatments (O, cyclic aerated subsurface drip irrigation; S, non-aerated subsurface drip irrigation as the control), three aeration levels (O1, O2, and O3, representing high, medium, and low aeration volume, respectively), and three fertilizer levels (F1, F2, and F3, representing high, medium, and low fertilizer amounts, respectively). Specifically, the high fertilizer treatment consisted of N 240 kg/hm^2^, P_2_O_5_ 120 kg/hm^2^, K_2_O 150 kg/hm^2^; the medium fertilizer treatment consisted of N 180 kg/hm^2^, P_2_O_5_ 90 kg/hm^2^, K_2_O 112.5 kg/hm^2^; the low fertilizer treatment consisted of N 120 kg/hm^2^, P_2_O_5_ 60 kg/hm^2^, K_2_O, 75 kg/hm^2^ [21].

The experiment was laid out following a randomized plot design with 12 trial treatments in total (Table 1). Each treatment was repeated in triplicate, resulting in a total of 36 plots. The single ridge and double rows planting method was adopted in the field plots, with each plot being 10 × 0.8 m. The depth of drip irrigation affects the chlorophyll content and dry matter accumulation of tomatoes [25]. Studies have reported that benefits were obtained when the drip irrigation of greenhouse tomato used lateral lines depths of 25 cm [26]. In the current study, lateral lines were laid at a depth of 25 cm with a dripper spacing of 33 cm. A total of 30 plants were planted on each ridge, and the ridge spacing was set at 10 × 0.8 m. To prevent lateral seepage, the ridges were separated by plastic film, and protective rows were established to mitigate any potential impact between treatments. The crops were planted and thoroughly irrigated on 22 September 2019, to ensure a high survival rate. The field management for all plots was consistent. It was reserved for topping after five spikes of fruit, and the entire growth period lasted approximately 140 days.

Irrigation scheduling was based on the evaporation value of an E601 evaporating dish placed in the greenhouse, with the amount applied being according to the 24 h evaporation at 08:00 of the previous day when the cumulative evaporation reached about 20 mm. It was calculated as I = A × KCP × EP, where I represents the irrigation amount of a single emitter (mL); A represents the plot area controlled by two branch pipes (m^2^), A = 0.8 m × 10 m = 8 m^2^; EP represents the accumulated evaporation in the interval between two irrigation events (mm), EP = 20 mm in the current experiment; KCP was the coefficient of the evaporating dish, (KCP = 1). 

The cyclic aeration device (Patent No.: cn103314697a) allowed for the integrated irrigation of water, fertilizer, and air. The maximum working pressure of the drip irrigation pipe used in the experiment was 0.2 MPa. Some researchers have demonstrated that as working pressure increases, the aeration proportion of water flow also increases. Furthermore, the volume of aeration can be regulated by adjusting the operating pressure [27].

Three pressure levels of 0.05 MPa, 0.1 MPa, and 0.15 MPa were configured create low, medium, and high aeration levels, respectively. The irrigation water was derived from deep groundwater and exhibited a relatively constant temperature of 18 °C. Under these specific water temperature conditions, three levels of aeration volume were designated as follows: high (O1) at 16.25%, medium (O2) at 14.58%, and low (O3) at 11.79%.

The oxygenation treatments involved the synchronous mixing of water and air through cyclic aeration before being introduced into the subsurface drip irrigation system, and then the water and gas mixture was transported to each crop’s root zone.

### 2.3. Determination Indices and Methods

#### 2.3.1. Quality Determination

Three fruits at the same stage of ripeness were chosen from each plant and homogenized for quality assessment. The total solids in the fruit were determined using a handheld sugar meter, while the soluble proteins were determined through Coomassie brilliant blue G-205 staining. The soluble sugar and titratable acid were measured using anthrone colorimetry and alkali titration, respectively, and the sugar–acid ratio was calculated as the ratio of soluble solid to titratable acid. Additionally, the vitamin C content was determined using the molybdenum blue colorimetric method [28,29].

#### 2.3.2. Measurement of Total Nitrogen, Phosphorus, and Potassium

The aboveground and underground parts of tomato plants were separately placed in an oven to remove moisture during the flowering and fruit-setting as well as fruit-expanding periods. Following drying, the tomato plants were ground using a grinder, sifted through a fine 0.5 mm sieve, and then isolated from air for preservation. The total nitrogen content was determined using the Kjeldahl method, the total phosphorus content was determined using the vanadium molybdenum yellow method, and the total potassium content was determined using the flame photometric method, which was computed using the following equations [30].

Nitrogen (phosphorus and potassium) absorption of each organ per plant = each organ total nitrogen (phosphorus and potassium) content per plant × dry matter mass of each organ per plant.

Distribution of nitrogen (phosphorus and potassium) in each organ (%) = absorption of nitrogen (phosphorus and potassium) in each organ/absorption of total nitrogen (phosphorus and potassium) in each plant × 100%.

#### 2.3.3. Determination of Fertilizer Absorption and Utilization Efficiency

The efficiency of fertilizer absorption and utilization during the flowering and fruit-setting periods, as well as during the fruit-expanding period, was calculated as follows.

Nitrogen (phosphorus, potassium) use efficiency (NUE) (kg/kg) = yield/total nitrogen (phosphorus, potassium) absorption [31]. 

Nitrogen (phosphorus, potassium) absorption efficiency (UPE) (kg/kg) = total nitrogen (phosphorus, potassium) absorption/nitrogen (phosphorus, potassium) nutrient input

Fertilizer partial productivity (PFP) (kg/kg) = total yield/fertilizer.

### 2.4. Data Analysis

Microsoft Excel was utilized for data sorting; SPSS Stat 29 was employed for data analysis. Analysis of variance (ANOVA) was utilized to comprehensively analyze fruit quality, absorption and distribution of fertilizer, as well as fertilizer and water use efficiency, considering the factors of aeration, fertilization levels, and their interactions (aeration × fertilization) to determine the significance of treatment differences. Origin 2024b was applied for data mapping. 

## 3. Results

### 3.1. Effects of Aerated Irrigation with Fertilizer on Greenhouse Tomato Quality

At the same aeration level, the contents of soluble solids of the tomatoes initially increased and then decreased as the amount of fertilizer increased (Table 2). The soluble solid content and sugar acid ratio contents in the tomatoes increased in proportion to the increase in aeration volume, while the same level of fertilizer was maintained. The soluble solid content in the high-aeration treatment increased by 15.63%, 17.83%, and 15.56% at the high, medium, and low fertilizer levels, respectively, compared to the corresponding non-aeration irrigation treatment. The sugar acid content in the high aeration treatment increased by 14.63%, 10.9%, and 7%, respectively; the VC content in the high aeration irrigation treatment increased by 22.39%, 39.15%, and 34.55% respectively; while under high, medium, and low fertilization levels, the soluble protein content under high-aeration irrigation treatments increased by 10.26%, 32.55%, and 42.41%, respectively, compared to the non-aeration irrigation treatment. The soluble sugar content of tomato fruit initially increased and then decreased as the aeration volume increased. Conversely, the organic acid content showed no discernible change under the same fertilizer level.

The analysis of variance revealed a highly significant impact of the aeration treatment on the soluble solid content, organic acid content, and sugar acid ratio of the tomatoes (*p* < 0.01). The fertilization treatment has a very significant impact on the organic acid content (*p* < 0.01).The soluble solid content has a significant impact (*p* < 0.05), but the sugar-acid ratio has no significant impact (*p* > 0.05). In single-factor analysis of variance, aeration treatment had a very significant impact on soluble VC content and soluble protein (*p* < 0.01), but had no significant impact on soluble sugar (*p* > 0.05).The application of fertilizer had a significant impact on the VC content (*p* < 0.05) but did not have a significant effect on the soluble sugar and soluble protein levels (*p* > 0.05). The interaction of the two factors only had a significant effect on the organic acid content (*p* < 0.05), but it did not have a significant effect on the soluble solid content or sugar acid ratio (*p* > 0.05). Aerated irrigation with fertilizer treatment did not yield any significant effect on the VC content, soluble sugar, or soluble protein (*p* > 0.05).

The range analysis results (Table 3) indicated that the tomatoes in the aerated treatment demonstrated higher soluble solid content, sugar acid ratio, vitamin C, soluble sugar, and soluble protein compared to those in the fertilizer treatment, while the effects on organic acid content were the same. The ratio of soluble solids, organic acids, and sugar acids were highest at high aeration irrigation levels and showed an increasing trend with the increase in aeration capacity. At the medium fertilizer level, the ratio of soluble solids, and sugar acids were the highest and exhibited a trend of initially increasing and then decreasing with the increase in aeration volume. The VC content of tomato fruit under high aeration irrigation and high fertilizer treatment reached the maximum value, and the VC content of fruit tended to increase with the increase of aeration amount and fertilization amount. The most suitable aeration level was highly aerated irrigation, and the most suitable fertilizer level was high fertilizer treatment. The optimal level of aeration for soluble sugar content was found to be medium aeration irrigation, followed by high aeration, and non-aerated irrigation. The level of soluble sugar initially increased and then decreased as the amount of fertilizer increased, with the highest level observed at the medium fertilization rate. The soluble protein content increased with the increase in aeration volume. The soluble protein content was found to be highest under high aerated irrigation, followed by medium aerated irrigation, then non-aerated irrigation. Specifically, the soluble protein content was the highest under high aerated irrigation with medium fertilizer treatment.

### 3.2. Effect of Aerated Irrigation and Fertilizer on Absorption and Distribution of Nitrogen, Phosphorus, and Potassium

#### 3.2.1. Effect of Aerated Irrigation and Fertilizer on Absorption and Distribution of Nitrogen

**Figure 1:**
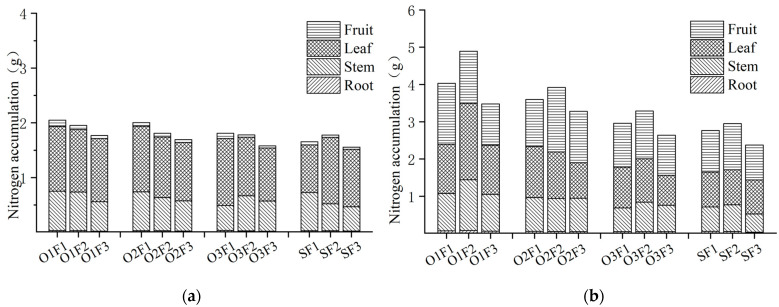
Total nitrogen content of greenhouse tomato in different plant parts under aerated irrigation with various fertilizer applications for different growing periods. (**a**) Flowering and fruit-setting period; (**b**) fruit-expanding period.

Nitrogen (N) accumulation increases with plant growth and development, reaching higher levels during the fruit-expanding period (FEP) compared to the flowering and fruit-setting period (BFP) (Figure 1). There were variations in the accumulation of nitrogen in different organs during both periods. During the flowering and fruit-setting period, the accumulation of nitrogen in various organs was found to be highest in the leaves, followed by in the stems, fruit, and roost. However, during the fruit-e-+xpanding period, the nitrogen ac cumulation was highest in the fruit, followed by in the leaves, stems, and roots.

Under the same level of fertilizer, the nitrogen accumulation under the aerated irrigation treatments increased with the increase in aeration in both periods. In the flowering and fruit-setting period, the nitrogen accumulation in the high, medium, and low aeration irrigation treatments under high fertilizer levels were 23.94%, 20.62%, and 9.37%, respectively, and those in the fruit-expanding period were 45.61%, 29.89%, and 6.87%, respectively, compared to the non-aerated irrigation treatments. At the same level of aeration, the accumulation of nitrogen in the other treatments increased during the flowering and fruit-setting period as fertilizer levels increased. However, it is worth noting that under the non-aerated irrigation treatment, the medium fertilizer amount resulted in higher nitrogen absorption compared to the high fertilizer amount. The in the high fertilizer treatment was 4.91% and 16.02% higher than that in the medium and low fertilizer treatments, respectively, under the high aeration level. During the fruit-expanding period, plant nitrogen accumulation initially increased and then decreased with the application of fertilizer. Under the same aeration level, the highest accumulation was observed in the medium fertilizer treatment, while the lowest accumulation was observed in the low fertilizer treatment. At the high aerated irrigation level, the nitrogen accumulation under medium fertilizer treatment increased by 21.37% and 40.56% compared to the high and low fertilizer treatments, respectively.

Range analysis revealed (Table 4) that the aeration treatment had a significant impact on the accumulation of nitrogen in the plants and fruits during the flowering and fruit-setting period (*p* < 0.05). However, it did not have a significant effect on the roots, stems, or leaves (*p* > 0.05). The application of fertilizer had a significant impact on the accumulation of nitrogen in the fruit (*p* < 0.05). However, it did not have a significant effect on the total nitrogen accumulation in the plant, or in the roots, stems, or leaves (*p* > 0.05). During the period of fruit swelling, there was a significant improvement in nitrogen accumulation in the plants (*p* < 0.01) and in the roots and leaves (*p* < 0.05) under aeration treatment but there was no significant effect on the stems (*p* > 0.05). Additionally, fertilization treatment had a significant effect on total plant nitrogen accumulation and fruit nitrogen accumulation (*p* < 0.05), but had no significant effect on root, stem, and leaf nitrogen accumulation (*p* > 0.05). The nitrogen accumulation was higher under the aeration treatment compared to the fertilizer treatment when considering the effect of both factors. There was no significant effect on nitrogen accumulation during the flowering and fruit-setting period under both factors (*p* > 0.05). However, there was a significant effect on nitrogen accumulation in the plants and fruits during the fruit-expanding period (*p* < 0.05).

#### 3.2.2. Phosphorus Absorption and Distribution Under Aerated Irrigation with Different Levels of Fertilizer Application

**Figure 2:**
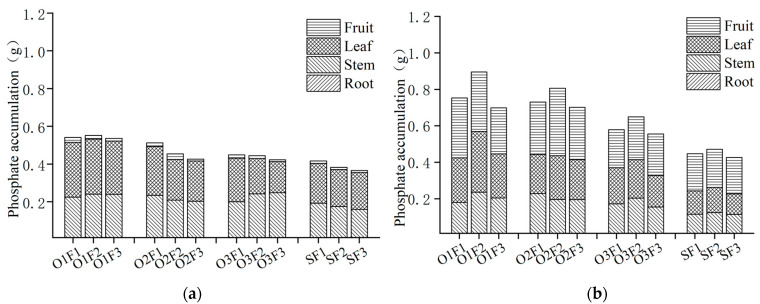
Total phosphorus content under aerated irrigation with different fertilizer applications of greenhouse tomato plant organs in different growing periods. (**a**) Flowering and fruit-setting period; (**b**) fruit-expanding period.

The impact of aerated irrigation with different fertilizer application levels on phosphorus (P) accumulation in various organs is depicted in Figure 2. Phosphorus accumulation increases as plants grow, with higher levels during the fruit-expanding period compared to the flowering and fruit-setting period. The highest levels of phosphorus content in the flowering and fruit-setting period were found in the leaves, followed by the stems, fruits, and roots. During the fruit-expanding period, the accumulation of phosphorus in each organ of the plant varied significantly. Except for the medium aerated irrigation with high fertilizer treatment, where the phosphorus accumulation in the stem exceeded that of the leaf, the order of accumulation was as follows: fruit > leaf > stem > root. Under the same level of fertilizer application, phosphorus accumulation during the flowering and fruit-setting period increased with higher aeration volume. The highest phosphorus content was observed in the high-aerated treatment with medium fertilizer application, while the lowest was found in the low-aerated irrigation with low fertilizer application treatment. Compared to the non-aerated treatments, phosphorus accumulation under medium fertilizer levels during the flowering and fruit-setting period were 44.24%, 18.85%, and 16.23% higher for high, medium, and low aeration irrigation treatments, respectively. Under the same level of aeration, the total phosphorus accumulation increased with increasing levels of fertilizer application. The total phosphorus accumulation in the plants under medium aerated irrigation with high fertilization was 20.18% higher than that under medium fertilizer treatment and 6.57% higher than that under low fertilizer treatment. The total phosphorus accumulation in the plant in the fruit-expanding period initially increased and then decreased with the amount of fertilization under the same aeration level. The highest accumulation was observed in the medium fertilizer treatment, while the lowest was found in the low fertilizer treatment. Specifically, at high aeration levels, the accumulation was 18.85% and 28.04% higher in the medium fertilizer treatment compared to the high and low fertilizer treatments, respectively.

The range analysis revealed (Table 5) that aeration treatment had a significant effect on the phosphorus accumulation in the plants, stems, and fruit in the flowering and fruit-setting period (*p* < 0.01) as well as nitrogen accumulation in the roots in the fruit-expanding period (*p* < 0.05). Fertilizer treatment did not have a significant effect on phosphorus accumulation in any organ during the flowering and fruit-setting period (*p* > 0.05). However, it did have a significant effect on the total phosphorus accumulation in plants and the nitrogen accumulation in the stems and fruit during the fruit-expanding period (*p* < 0.05). Phosphorus accumulation was found to be higher under aerated irrigation treatment compared to fertilizer treatment, taking into account the impact of both factors. The interaction between aeration treatment and fertilizer treatment did not have a significant effect on the nitrogen accumulation during the flowering and fruit-setting period (*p* > 0.05), but it did have a significant effect on the total nitrogen accumulation in the plants, as well as in the stems and fruit during the fruit-expanding period (*p* < 0.05), while it had no significant effect on the other plant organs (*p* > 0.05).

#### 3.2.3. Potassium Absorption and Distribution Under Aerated Irrigation with Different Fertilizer Application Levels

**Figure 3:**
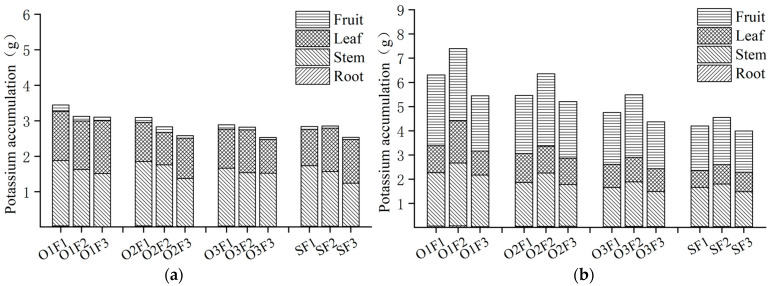
Total potassium content under aerated irrigation with different fertilizer application levels of greenhouse tomato organs for different growing periods. (**a**) Flowering and fruit-setting period; (**b**) fruit-expanding period.

The accumulation of potassium (K) increased with plant growth, reaching higher levels during the fruit-expanding period compared to the flowering and fruit-setting period (Figure 3). The highest accumulation of potassium was detected in the stem, followed by the leaf, fruit, and root during the flowering and fruit-setting period. There was significant variation in the potassium accumulation in each organ of the plant during the fruit-expanding period, with the highest value observed in the fruit, followed by the stem, leaf, and root. At the same level of fertilizer application during the flowering and fruit-setting period, it was observed that, with the exception of the medium fertilizer treatment, potassium accumulation under low aerated irrigation was lower than under non-aerated irrigation. In contrast, the other treatments showed an increase in potassium accumulation with increasing aeration volume. The highest potassium content was detected under high aerated irrigation with high fertilizer treatment, while the lowest accumulation appeared in the low aerated irrigation with low fertilizer treatment. The high aerated irrigation treatment resulted in an increase in 21.36%, 9.53%, and 22.39% under high, medium, and low fertilizer levels, respectively, compared to the non-aerated irrigation. Under the same level of fertilizer during the fruit-expanding period, the total accumulation of potassium increased with the rise in aeration volume. Under high, medium, and low fertilizer levels, the total accumulation of potassium in the high aerated irrigation treatment exceeded that of the non-aerated irrigation treatment by 50.1%, 62.53%, and 36.49%, respectively.

At the same level of aeration, potassium absorption increased with the application of increasing levels of fertilizer during the flowering and fruit-setting period. Specifically, compared to the low fertilizer treatment, the accumulation of potassium was 11.07%, 19.81%, and 14.09% higher under high, medium, and low aerated irrigation in the high fertilizer treatment, respectively. At the same level of aeration, potassium accumulation initially increased and then decreased with the increase in fertilizer amount in the fruit-expanding period. The highest and lowest values of potassium accumulation were observed in the medium and low fertilizer treatments, respectively. Specifically, compared to the low fertilizer treatment, potassium accumulation was 15.75%, 4.96%, and 8.73% higher under high, medium, and low aerated irrigation in the medium fertilizer treatment, respectively.

The range analysis revealed (Table 6) that the potassium accumulation in the plants, stems, and fruits significantly improved under aeration treatment during the flowering and fruit-setting period (*p* < 0.05). However, there was no significant impact found on the roots or leaves (*p* > 0.05). On the other hand, the fertilizer treatment had a significant impact on the potassium accumulation in stems (*p* < 0.05), while no apparent difference was found in the total potassium accumulation of plants, roots, leaves, and fruits (*p* > 0.05). During the fruit-expanding period, aeration treatment had an extremely significant effect on the total nitrogen accumulation of plants, stems, and fruits (*p* < 0.01), while it had a significant effect on the roots and leaves (*p* < 0.05). The fertilization treatment has a significant effect on the total potassium accumulation of plants, leaves, and fruits (*p* < 0.05), while no apparent difference was found in the roots or stems (*p* > 0.05). The accumulation of potassium was higher under the aerated treatment compared to the fertilizer treatment, taking both factors into consideration. The interaction between aeration treatment and fertilizer treatment had a significant effect on the potassium accumulation in the leaves and fruits during the fruit-expanding period (*p* < 0.05) but did not have a significant effect on any organ during the flowering and fruit-setting period (*p* > 0.05).

### 3.3. Fertilizer Absorption Efficiency Under Aerated Irrigation with Different Fertilizer Application

The absorption efficiency (UPE) of nitrogen, phosphorus, and potassium during the flowering and fruit-setting period was found to be higher than during the fruit-expanding period (Table 7). Furthermore, it was observed that the nitrogen absorption efficiency under aerated treatment exceeded that of non-aerated irrigation treatment. Under the same level of fertilizer application, it was observed that the nitrogen absorption efficiency of medium aerated irrigation during the flowering and fruit-setting period was lower compared to low aerated irrigation under medium fertilizer application. Additionally, the nitrogen and phosphorus absorption efficiency of high aeration treatment during the fruit-expanding period was lower than that of medium aeration treatment under low fertilizer irrigation. However, it is worth noting that the nitrogen absorption efficiency in the other treatments increased with an increase in aeration. Under consistent aeration levels, except for the medium aeration treatment during the flowering and fruit-setting period, as well as the high and medium aeration treatment during the fruit-expanding period, nitrogen absorption efficiency decreased with an increase in fertilizer application across all other treatments. Furthermore, phosphorus and potassium absorption efficiency also declined with increasing rates of fertilizer application. During the flowering and fruit-setting period, the highest absorption efficiency of nitrogen and phosphorus was observed under high aerated irrigation with low fertilizer treatment, while the lowest efficiency was detected for non-aerated irrigation with high fertilizer treatment. In terms of potassium absorption efficiency, it was found to be the highest under medium aeration with low fertilizer treatment and lowest under low aeration with high fertilizer treatment, showing a notable difference of 1.19 kg·kg^−1^ between the two conditions. During the fruit-expanding period, the highest absorption efficiency of nitrogen, phosphorus, and potassium was observed with medium aerated irrigation under low fertilizer treatment. Conversely, the lowest absorption efficiency was found with non-aerated irrigation under high fertilizer treatment, with differences of 0.47 kg·kg^−1^, 0.27 kg·kg^−1^, and 1.8 kg·kg^−1^, respectively.

The analysis of variance revealed that aerated irrigation significantly enhanced the efficiency of nitrogen and potassium absorption during the flowering and fruit-setting period (*p* < 0.05), while it had no significant effect on phosphorus absorption efficiency (*p* > 0.05). Furthermore, all three nutrient absorption efficiencies were found to be improved during the fruit-expanding period (*p* < 0.05). Fertilizer application had an extremely significant effect on nitrogen and potassium absorption efficiency (*p* < 0.01) and a significant effect on phosphorus absorption efficiency during the flowering and fruit-setting period (*p* < 0.05). During the fruit-expanding period, it had an extremely significant effect on all three nutrient absorption efficiencies (*p* < 0.01). During the flowering and fruit-setting period, there was a significant effect on potassium absorption efficiency (*p* < 0.05), while no significant effects were observed on nitrogen and phosphorus when considering the interaction of the two factors (*p* > 0.05). During the fruit-expanding period, there were significant effects on all three nutrients (*p* < 0.05).

### 3.4. Fertilizer and Water Use Efficiency Under Aerated Irrigation with Different Fertilizer Application

The nutrient use efficiency (NUE) and water use efficiency (WUE) under aerated irrigation with different fertilizer applications are presented in Table 8. The nitrogen and phosphorus use efficiency of in high and medium aerated irrigation treatments were found to be higher than that in the non-aerated irrigation treatment. Conversely, the efficiency in the low aerated irrigation treatment was lower compared to the non-aerated irrigation treatment under the low fertilizer level. Under the same fertilizer level, nitrogen and phosphorus increased first and then decreased with the increase in aeration. Under the same aeration irrigation level, the phosphorus use efficiency increased first and then decreased with the increase in fertilizer. Except for the nitrogen use efficiency in the high aeration irrigation and high fertilizer treatment being lower than that in the high aeration irrigation and low fertilizer treatment, the nitrogen use efficiency of the other treatments increased first and then decreased with the increase in fertilizer, the nitrogen and phosphorus utilization efficiencies were found to be highest with medium aerated irrigation, while it was lowest with low aerated irrigation. Interestingly, the potassium utilization efficiency did not follow the same pattern as nitrogen and phosphorus. In fact, the potassium utilization efficiency was highest in the medium fertilizer treatment under high aerated irrigation and lowest in the low fertilizer treatment under non-aerated irrigation.

Both the partial factor productivity of the fertilizer and water use efficiency in the cyclic aeration irrigation treatment was higher than that in the non-aeration irrigation treatment. Under the same level of fertilization. The highest values were observed in the high aerated irrigation treatment, while the lowest values were seen under low aerated irrigation treatment. The partial factor productivity of fertilizer under high aerated irrigation treatment was found to be 20.77%, 21.05%, and 35.18% higher than that of non-aerated irrigation, respectively. Additionally, the water use efficiency in the high aerated irrigation treatment was observed to be 12.06%, 22.08%, and 14.09% higher than that in the non-aerated treatment, respectively. Under the same level of aeration, the partial factor productivity of fertilizer gradually decreased with increasing fertilizer application. The lowest partial factor productivity of fertilizer was observed in the high fertilizer treatment, while the highest was seen in the low fertilizer treatment. Additionally, water use efficiency in the medium fertilizer treatment proved to be most effective, exhibiting a 11.65% increase compared to that in the high fertilizer treatment and a 16.71% increase compared to that in the low fertilizer treatment.

## 4. Discussion

The findings of this study demonstrate that aerated subsurface drip irrigation can significantly enhance the quality of greenhouse tomatoes, such as the contents of soluble solids, organic acids, sugar acid ratio, vitamin C, soluble sugar, and soluble protein, which is consistent with previous findings [22,32]. Studies have reported that compared with non-aerated treatments, aerated drip irrigation significantly increased the vitamin C content, sugar-acid ratio, and soluble solids content of tomatoes [33]. When crops are grown under aerated drip irrigation, enhanced oxygen content in the rhizosphere is observed [34], which helps with alleviating hypoxia conditions and improving root respiration [17]. The improved root condition contributes to an improvement in leaf photosynthetic performance, among which photosynthetic carbon assimilation products significantly improve fruit quality [35]. These observations agree with those of studies on cantaloupe [36] and cucumber [37]. Fruit quality indicators, such as soluble solids, sugar–acid ratio, vitamin C, and soluble sugar, were found to be higher under medium fertilizer application compared to those under low and high fertilizer treatments. Both low and high levels of fertilizer application inhibited the growth of tomato plants. The improved growth of tomato seedlings and fruit quality under the medium fertilizer application in our study confirms the findings of Yang et al. [38], who concluded that the appropriate amount of fertilizer can promote the nutritional growth and yield of watermelon, improve photosynthetic efficiency, and quality. It was also confirmed by Yao et al. [39] that the sugar content indicators of watermelon under reduced chemical fertilizer were better than those with conventional fertilizer application. The study conducted by Fen Wang et al. [40] indicated that the combination of thed60% traditional nitrogen application rate and DMPP treatment has the most significant effect on increasing the anthocyanin and soluble sugar content in apple fruits. 

As the three most essential nutrients for plant growth and development [40], nitrogen is closely associated with fruit volume, arils quantity, and fruit weight [20]. Phosphorus plays a vital role in plant respiration and metabolism, promoting root growth as well as cold and drought resistance. Additionally, potassium contributes to the synthesis of sugar, cellulose, and lignin while enhancing the lodging resistance of plants [41]. The accumulation of nitrogen, phosphorus, and potassium in plants grown under aerated irrigation increased compared with that in non-aerated irrigation treatment, and the accumulation showed a positive correlation with the volume of aeration, which aligns with the research findings of Lei et al. [42]. The increased root absorption capacity was due to the ambient soil aeration, which resulted in the efficient uptake of nutrients and water use [43,44,45]. The optimal application of fertilizer for greenhouse tomatoes varies at different stages. During the flowering and fruit-setting period, the absorption of nitrogen, phosphorus, and potassium increased with increasing levels of fertilizer application, while in the fruit-expanding period, the accumulation initially increased and then decreased, reaching its maximum under medium fertilizer treatment. This could be attributed to the continuous application of fertilizers during the fruit swelling stage, resulting in an excessive amount of nutrients that hinder the absorption of essential nutrients [46].

In an agricultural system, both the plant itself and its rhizosphere environment are critical factors that impact the efficient use of nutrients, such as nitrogen, phosphorus, and potassium [40]. The presence of hypoxia in the rhizosphere inhibits the aerobic respiration of crop roots and microorganisms. This hinders soil microorganisms from decomposing nutrients through aerobic respiration, consequently affecting the absorption and utilization of soil nutrients [34]. The nitrogen, phosphorus, and potassium absorption and utilization efficiency in the aerated irrigation treatment were generally higher compared to those in the non-aerated treatment. This increase in efficiency was observed to be related to the aeration volume. These findings are consistent with those of Zhu et al. [47], who reported that the soil total phosphorus and total potassium contents under the 75% aeration volume and late-stage aeration drip irrigation treatment were the smallest, which were 42.86% and 14.82% lower than in the control, respectively; the 50% aeration volume and late-stage aeration drip irrigation treatment had the lowest total nitrogen content. This may have been due to the fact that the soil enzyme and microbial activity improved due to the increased soil aeration through aerated irrigation [34], which contributed to the creation of a more favorable microenvironment around the rhizosphere [48] and improved the efficient utilization of nutrients.

Moderate fertilization plays a key role in enhancing crop yield, promoting nutrient absorption and improving water use efficiency [49]. Excessive fertilization, in addition to damaging the soil structure, reduces water and fertilizer utilization efficiency [50]. Excessive application of nitrogen fertilizer increases the nitrate nitrogen concentration in groundwater, which is detrimental to the sustainable development of agricultural production [51]. The nitrogen, phosphorus, and potassium absorption efficiencies of greenhouse tomatoes all exhibited a decreasing trend with increased fertilizer application, while the utilization efficiency initially increased and then decreased with the rise in fertilizer application. The evidence fully demonstrated the fact that excessive fertilization did not lead to an improvement in the fertilizer absorption efficiency. The high aerated and medium fertilizer treatment proved to be the most effective coupling for greenhouse tomatoes under the experimental conditions. This conclusion takes into account various factors including plant growth characteristics, fruit quality, fertilizer absorption and utilization efficiency, as well as water use efficiency in all treatments.

## 5. Conclusions

1. Cyclic aeration under subsurface drip irrigation system enhanced fruit quality. In particular, the fruit’s soluble solids, organic acids, sugar acid ratio, VC, and soluble protein contents were found to be higher under high aeration and medium fertilizer treatment than in the other treatments. The highest soluble sugar content was achieved under medium aeration and medium fertilizer treatment.
2. The cyclic aeration with subsurface drip irrigation systems enhanced the uptake and accumulation of nutrients, as well as the nutrient uptake efficiency of tomato plants. Under the same level of fertilizer application, the cumulative absorption of nitrogen, phosphorus, and potassium was found to be highest in the high aeration irrigation treatment, while it was lowest in the non-aerated irrigation treatment. The efficiency of nitrogen, phosphorus, and potassium absorption increased with the aeration volume during both growing periods. Compared to the non-aerated irrigation treatment, the high aeration irrigation treatment resulted in an increase in 35.71%, 28.57%, and 36.21% in nitrogen, phosphorus, and potassium absorption, respectively. In the flowering and fruit-setting period, the accumulation of nitrogen and potassium increased with the application of fertilizer under the same aeration level. The accumulation of phosphorus initially increased and then decreased. For the fruit-expanding period, the accumulation of all three nutrients initially increased and then decreased with increasing fertilizer application. Compared to the fertilizer treatment, nutritional accumulation was higher in the aerated treatment when both factors were combined.
3. The aerated treatment demonstrated higher fertilizer and water use efficiency compared to the non-aerated irrigation treatment. The efficiency in the utilization of all three nutrients initially increased and then decreased with the application of increasing amounts of fertilizer. The medium fertilizer treatment demonstrated the highest level of efficiency. The highest nitrogen, phosphorus, potassium and water use efficiencies were detected under high aerated irrigation with medium fertilizer application. The increases were 6.68%, 8.48%, 19.34%, and 22.08% for nitrogen, phosphorus, potassium and water use efficiency, respectively. The highest partial factor productivity of fertilizer was observed under high aerated irrigation with low fertilizer treatment, while the lowest was recorded under non-aerated irrigation with high fertilizer application. The partial factor productivity of fertilizer and potassium use efficiency showed significant improvement under the interaction of the two factors (*p* < 0.05), while nitrogen, phosphorus, and water use efficiency did not have a significant effect (*p* > 0.05).
4. The high aeration and medium fertilizer treatment proved to be the most effective coupling for greenhouse tomatoes under the experimental conditions. This conclusion takes into account various factors including plant growth characteristics, fruit quality, fertilizer absorption and utilization efficiency, as well as water use efficiency of all treatments.

## Figures and Tables

**Table 1:** Schematic representation of the experimental setup including all treatments.

Treatment	Irrigation Method	Fertilizer Level	Treatment	Irrigation Method	Fertilizer Level
O1F1	High aeration volume	High fertilizer amount	O3F1	Low aeration volume	High fertilizer amount
O1F2	High aeration volume	Medium fertilizer amount	O3F2	Low aeration volume	Medium fertilizer amount
O1F3	High aeration volume	Low fertilizer amount	O3F3	Low aeration volume	Low fertilizer amount
O2F1	Medium aeration volume	High fertilizer amount	SF1	No aeration	High fertilizer amount
O2F2	Medium aeration volume	Medium fertilizer amount	SF2	No aeration	Medium fertilizer amount
O2F3	Medium aeration volume	Low fertilizer amount	SF3	No aeration	Low fertilizer amount

**Table 2:** Fruit quality under aerated irrigation with various fertilizer applications.

Treatment		Soluble Solids (%)	Organic Acid (%)	Ratio of Sugar to Acid (%)	Vitamin C (mg·100 g^−1^)	Soluble Sugar (%)	Soluble Protein (mg·g^−1^)
O1F1		7.40 ± 0.29 ab	0.54 ± 0.01 abc	13.79 ± 0.43 a	23.72 ± 1.83 a	4.13 ± 0.45 ab	3.01 ± 0.72 abc
O1F2		7.60 ± 0.35 a	0.55 ± 0.01 a	13.84 ± 0.76 a	24.95 ± 1.21 a	4.31 ± 0.49 ab	3.38 ± 0.26 a
O1F3		7.13 ± 0.41 abcd	0.52 ± 0.02 cde	13.58 ± 0.62 a	22.94 ± 1.84 abc	3.87 ± 0.39 ab	3.19 ± 0.66 ab
O2F1		7.33 ± 0.37 abc	0.54 ± 0.01 abc	13.65 ± 0.80 a	23.46 ± 1.92 ab	4.20 ± 0.24 ab	2.95 ± 0.46 abc
O2F2		7.53 ± 0.32 a	0.55 ± 0.01 ab	13.77 ± 0.61 a	21.39 ± 1.22 abcd	4.53 ± 0.17 a	3.11 ± 0.15 abc
O2F3		6.62 ± 0.41 de	0.52 ± 0.02 e	12.8 ± 0.45 ab	19.68 ± 1.77 cde	3.93 ± 0.07 ab	2.79 ± 0.38 abc
O3F1		6.68 ± 0.43 cde	0.53 ± 0.01 abcd	12.52 ± 0.83 ab	20.00 ± 2.22 bcde	3.76 ± 0.35 ab	2.59 ± 0.40 abc
O3F2		6.8 ± 1.13 bcde	0.54 ± 0.01 ab	12.52 ± 2.08 ab	19.79 ± 1.09 bcde	4.00 ± 0.85 ab	2.64 ± 0.61 abc
O3F3		6.57 ± 0.21 de	0.52 ± 0.01 de	12.63 ± 0.46 ab	17.83 ± 1.91 de	3.64 ± 0.34 ab	2.38 ± 0.28 bc
SF1		6.40 ± 0.28 de	0.53 ± 0.02 bcd	12.03 ± 0.72 b	19.38 ± 1.00 cde	3.58 ± 0.56 b	2.73 ± 0.38 abc
SF2		6.45 ± 0.72 de	0.52 ± 0.01 e	12.48 ± 1.40 ab	17.93 ± 1.87 de	3.94 ± 0.7 ab	2.55 ± 0.38 abc
SF3		6.17 ± 0.28 e	0.49 ± 0.01 f	12.7 ± 0.61 ab	17.05 ± 1.22 e	3.71 ± 0.33 ab	2.24 ± 0.14 c
F value	Aeration volume (A)	12.76 **	15.7 **	7.09 **	15.07 **	2.19 ns	4.98 **
	Fertilizer amount (F)	4.61 *	34.63 **	0.3 ns	4.19 *	2.38 ns	1.12 ns
	A × F	0.55 ns	3.05 *	0.68 ns	1.54 ns	0.15 ns	0.33 ns

Note: values followed by different small letters in the same column indicate significant differences at 0.05. * indicates *p* < 0.05 and ** indicates *p* < 0.01, ns indicates *p* > 0.05.

**Table 3:** Range analysis of fruit quality under aerated irrigation with various fertilizer applications.

Factor	Soluble Solids (%)	Organic Acid (%)	Ratio of Sugar to Acid (%)	Vitamin C (mg·100 g^−1^)	Soluble Sugar (%)	Soluble Protein (mg·g^−1^)
O1	7.38	0.54	13.74	23.87	4.1	3.19
O2	7.16	0.53	13.4	21.51	4.22	2.95
O3	6.68	0.53	12.55	19.2	3.8	2.54
S	6.34	0.51	12.4	18.12	3.75	2.51
range	1.04	0.03	1.34	5.75	0.47	0.68
F1	6.95	0.54	13.00	21.64	3.92	2.82
F2	7.10	0.54	13.15	21.02	4.2	2.92
F3	6.62	0.51	12.92	19.38	3.79	2.65
range	0.48	0.03	0.23	2.26	0.41	0.27

**Table 4:** Total nitrogen content under aerated irrigation with different fertilizer applications for different growing periods.

Treatment	Nitrogen Content (G)									
	Plant		Root		Stem		Leaf		Fruit	
	BEP	FEP	BEP	FEP	BEP	FEP	BEP	FEP	BEP	FEP
Aeration volume (A)	*	**	ns	*	ns	ns	ns	*	*	**
O1	1.924 a	4.133 a	0.031 a	0.070 a	0.653 a	1.050 a	1.292 a	1.498 a	0.081 a	1.311 ab
O2	1.836 ab	3.599 a	0.030 a	0.056 a	0.620 a	0.895 a	0.990 a	1.189 ab	0.061 ab	1.459 a
O3	1.723 b	2.962 b	0.030 a	0.051 a	0.548 ab	0.711 ab	1.021 a	0.988 b	0.058 ab	1.045 b
S	1.662 b	2.697 bc	0.030 a	0.050 a	0.543 ab	0.620 ab	1.127 a	0.830 b	0.045 b	1.098 b
Fertilizer amount (F)	ns	*	ns	ns	ns	ns	ns	ns	*	*
F1	1.880 a	3.337 a	0.034 a	0.060 a	0.644 a	0.799 a	1.119 a	1.180 a	0.082 a	1.221 ab
F2	1.830 a	3.763 ab	0.033 a	0.062 a	0.608 a	0.938 a	1.095 a	1.251 a	0.056 b	1.388 a
F3	1.649 b	2.943 bc	0.024 ab	0.049 a	0.520 ab	0.719 a	1.109 a	0.948 a	0.046 b	1.075 b
A × F	ns	*	ns	ns	ns	ns	ns	ns	ns	*

Note: BFP and FEP represent flowering and fruit-setting period and fruit-expanding period, respectively; the same applies below. Values followed by different small letters in the same column indicate significant differences at 0.05. * indicates *p* < 0.05 and ** indicates *p* < 0.01, ns indicates *p* > 0.05.

**Table 5:** Total phosphorus content under aerated irrigation with different fertilizer application levels in different growing periods.

Treatment	Phosphate Content (G)									
	Plant		Root		Stem		Leaf		Fruit	
	BEP	FEP	BEP	FEP	BEP	FEP	BEP	FEP	BEP	FEP
Aeration volume (A)	*	**	ns	*	*	**	ns	*	*	**
O1	0.136 a	0.782 a	0.005 a	0.009 a	0.229 a	0.198 a	0.295 a	0.256 a	0.020 a	0.303 a
O2	0.116 ab	0.746 a	0.006 a	0.009 a	0.209 ab	0.195 a	0.216 a	0.224 a	0.020 a	0.328 a
O3	0.110 ab	0.594 b	0.006 a	0.008 a	0.224 a	0.186 ab	0.194 a	0.194 ab	0.014 ab	0.262 ab
S	0.097 bc	0.448 bc	0.006 a	0.006 ab	0.183 b	0.111 bc	0.247 a	0.116 b	0.012 b	0.204 b
Fertilizer amount (F)	ns	*	ns	ns	ns	*	ns	ns	ns	*
F1	0.120 a	0.627 ab	0.007 a	0.008 a	0.216 a	0.158 ab	0.248 a	0.183 a	0.019 a	0.257 a
F2	0.115 a	0.705 a	0.006 a	0.008 a	0.210 a	0.187 a	0.235 a	0.215 a	0.019 a	0.314 a
F3	0.110 a	0.595 ab	0.005 a	0.008 a	0.208 a	0.172 a	0.232 a	0.194 a	0.011 a	0.251 a
A × F	ns	*	ns	ns	ns	*	ns	ns	ns	*

Note: values followed by different small letters in the same column indicate significant differences at 0.05. * indicates *p* < 0.05 and ** indicates *p* < 0.01, ns indicates *p* > 0.05.

**Table 6:** Total potassium content under aerated irrigation with different fertilizer application levels in different growing periods.

Treatment	Potassium Content (G)									
	Plant		Root		Stem		Leaf		Fruit	
	BEP	FEP	BEP	FEP	BEP	FEP	BEP	FEP	BEP	FEP
Aeration volume (A)	*	**	ns	*	*	**	ns	*	*	**
O1	3.222 a	1.596 a	0.029 a	0.058 a	1.512 bc	2.311 a	1.413 a	1.285 a	0.134 a	2.728 a
O2	2.833 b	1.419 b	0.032 a	0.053 a	1.394 bc	2.213 a	1.053 ab	1.056 ab	0.120 a	2.743 a
O3	2.743 bc	1.218 bc	0.029 a	0.042 b	2.345 a	1.772 b	1.038 ab	0.970 ab	0.083 ab	2.089 b
S	2.741 bc	1.062 c	0.028 a	0.041 b	1.854 b	1.342 bc	1.159 a	0.636 b	0.066 b	1.795 bc
Fertilizer amount (F)	ns	*	ns	ns	*	ns	ns	*	ns	*
F1	3.063 a	1.295 ab	0.036 a	0.048 a	1.922 a	1.814 a	1.154 a	0.936 ab	0.125 a	2.226 ab
F2	2.908 a	1.487 a	0.031 a	0.053 a	1.866 a	2.001 a	1.141 a	1.066 a	0.108 a	2.601 a
F3	2.684 ab	1.189 b	0.022 ab	0.044 a	1.541 ab	1.913 a	1.202 a	0.958 ab	0.069 a	2.188 ab
A × F	ns	ns	ns	ns	ns	ns	ns	*	ns	*

Note: values followed by different small letters in the same column indicate significant differences at 0.05. * indicates *p* < 0.05 and ** indicates *p* < 0.01, ns indicates *p* > 0.05.

**Table 7:** Fertilizer absorption efficiency under aerated irrigation with different fertilizer applications in different growing periods.

Treatment		N UPE/(kg·kg^−1^)		P UPE/(kg·kg^−1^)		K UPE/(kg·kg^−1^)	
		BEP	FEP	BEP	FEP	BEP	FEP
O1F1		0.64 cd	0.5 abc	0.38 b	0.18 bcde	1.72 bc	1.26 bc
O1F2		0.81 b	0.81 a	0.46 ab	0.30 ab	2.08 abc	1.97 ab
O1F3		1.35 a	0.72 ab	0.69 a	0.35 a	2.7 ab	2.18 ab
O2F1		0.63 cd	0.45 bc	0.32 bc	0.17 bcde	1.61 c	1.05 c
O2F2		0.67 cd	0.65 abc	0.36 b	0.27 abcd	1.75 bc	1.7 bc
O2F3		0.93 b	0.82 a	0.51 ab	0.38 a	2.78 a	2.64 a
O3F1		0.57 d	0.35 c	0.28 bc	0.14 cde	1.59 c	0.87 c
O3F2		0.74 bc	0.51 abc	0.37 b	0.27 abcd	1.98 abc	1.57 bc
O3F3		0.86 b	0.66 abc	0.53 ab	0.28 abc	2.66 ab	1.75 bc
SF1		0.52 d	0.35 c	0.28 bc	0.11 e	1.72 bc	0.84 c
SF2		0.76 bc	0.44 bc	0.38 b	0.13 de	1.9 abc	0.87 c
SF3		0.84 b	0.59 abc	0.55 ab	0.24 abcde	2.2 abc	1.6 bc
F value	Aeration volume (A)	3.94 *	3.43 *	2.75 ns	4.48 *	4.46 *	4.54 *
	Fertilizer amount (F)	5.52 **	9.03 **	4.55 *	13.44 **	8.11 **	14.07 **
	A × F	1.28 ns	3.49 *	0.81 ns	2.57 *	2.72 *	2.87 *

Note: values followed by different small letters in the same column indicate significant differences at 0.05. * indicates *p* < 0.05 and ** indicates *p* < 0.01, ns indicates *p* > 0.05.

**Table 8:** Nutrient uptake efficiency under aerated irrigation with different fertilizer application levels in different growing periods.

Treatment		N NUE (kg·kg^−1^)	P NUE (kg·kg^−1^)	K NUE (kg·kg^−1^)	PFP (kg·kg^−1^)	Water Use Efficiency (kg·m^−3^)
O1F1		266.80 abc	1528.69 b	163.55 bc	98.11 f	29.96 ± 1.71 b
O1F2		291.96 ab	1637.97 ab	191.21 a	128.25 d	33.45 ± 2.56 a
O1F3		278.24 ab	1142.55 cd	167.40 bc	171.06 a	28.66 ± 2.70 bc
O2F1		283.81 ab	1473.12 b	182.23 b	87.42 f	29.08 ± 2.76 b
O2F2		347.19 a	1855.71 a	154.17 bcd	112.97 e	28.49 ± 2.06 bc
O2F3		246.08 bc	1160.06 cd	149.24 d	164.67 ab	26.8 ± 0.84 bcd
O3F1		247.39 bc	1148.44 cd	169.78 bc	85.23 f	27.57 ± 2.16 bcd
O3F2		297.83 ab	1172.41 cd	145.57 cd	113.69 e	27.8 ± 3.25 bcd
O3F3		206.71 cd	893.32 d	157.09 bc	150.57 c	25.79 ± 1.25 cd
SF1		242.49 bc	1385.59 bc	149.25 bc	81.24 f	26.75 ± 2.04 bcd
SF2		273.69 ab	1509.95 b	160.21 bc	105.95 e	27.4 ± 0.76 bcd
SF3		226.72 bcd	1097.49 cd	139.34 cd	126.54 bc	25.12 ± 2.77 d
F value	Aeration volume (A)	3.94 *	2.75 ns	4.46 *	7.95 **	10.84 **
	Fertilizer amount (F)	5.52 **	4.55 *	8.11 **	10.63 **	7.63 **
	A × F	1.28 ns	0.71 ns	2.72 *	3.24 *	0.91ns

Note: values followed by different small letters in the same column indicate significant differences at 0.05. * indicates *p* < 0.05 and ** indicates *p* < 0.01, ns indicates *p* > 0.05.

## Data Availability

Date will be made available on request. The data are not publicly available due to privacy.
